# 

**Published:** 1898-05-21

**Authors:** 


**Notices of Births, Deaths, and Marriages** are inserted in this column at a charge of 2s. 6d. for an announcement not exceeding four lines.

### NOTICE TO CORRESPONDENTS.

All MS., Letters, Books for Review, and other matters intended for the Editor should be addressed The Editor, "The Hospital," The Hospital Building, 28 & 29, Southampton Street, Strand, W.C.

The Editor cannot undertake to return rejected MS., even when accompanied by stamped directed envelope.

**Notice to Advertisers and Subscribers.**

All Advertisements, Orders for Copies of The Hospital, and Business Communications should be addressed to **THE MANAGER, (Not to The Editor),** The Hospital, The Hospital Building, 28 & 29, Southampton Street, Strand, London, W.C.

The Cost of Subscription, post free, is as follows:—Per week, 2½d. per quarter, 2s. 8d.; per half-year, 5s. 3d.; per annum, 10s. 6d. Foreign:—Per annum, 15s. 2d.; payable, in all cases, in advance.

**NOTICE.—Vol. XXIII. of "The Hospital" (October, 1897, to March, 1898), in handsome cloth binding, is now on sale at the Office of this Journal, price 7s. 6d. Cases for binding the Numbers contained in this Volume, 1s. 6d. Index to Vol. XXIII., 2½d., post free.**

**The Monthly Part for June will be ready on June 1. Price 6d., by post 8d.**

### BOOKS RECEIVED.

Swan Sonnenschein and Co.

"A Century of Vaccination," By W. Scott Tebb, M.A., M.D., D.P.H.

W. H. and L. Collingridge.

"The City of London Directory, 1898."

The Boston City Hospital.

Medical and Surgical Reports.

**Periodicals, &c.**—Literary Digest, New Age, Woman's Signal, Industries and Iron, The Critic, Boy's Own Paper, Sunday at Home, English Illustrated Magazine, Electrical Review, London, Medical Record, Asylum News, National Hospital Record, Councils' Gazette, Medical Press, Medical News, Health, Public Health Journal.

### SCRAPS AND GLEANINGS.

THE Chelmsford and Essex Infirmary received in 1897 £1,417, and expended £1,566.

IT is proposed to hold an Eisteddfod at Swansea this year in aid of the Swansea General Hospital.

THE number of patients admitted to the Beau Site Convalescent Home at Hastings during 1897 was 292.

ONE HUNDRED AND THIRTY cases were treated at the Teddington Cottage Hospital during the year 1897.

THE proposed new fever hospital for the Borough of Reigate is to contain accommodation for about 25 patients.

UNDER the will of the late Mr. Charles Wheeley Lea the Worcester Infirmary has received a legacy of £[illegible]0,000.

A COMMITTEE has been appointed to consider the suggested enlargement of the Montagu Hospital at Mexboro'.

SEVENTY patients, of whom one-half were under 16 years of age, were admitted to the Hinckley Cottage Hospital in 1897.

THERE was a daily average occupation of 48 beds, out of a total of 50 provided, at the Bradford Children's Hospital in 1897.

IT has been decided that the one wing of the new cottage hospital at Clacton shall be commenced. The cost will be about £900.

THE average cost per week for a patient at the Cumberland and Westmoreland Convalescent Institution at Silloth during 1897 was 10s. 2⅜d.

SEVENTY-FIVE men and 69 women, or a total of 144 patients, were admitted to the Hahnemann Convalescent Home at Bournemouth in 1897.

THE Hospital Saturday Collection made in aid of the Wolverhampton General Hospital in 1897 amounted to £3,870, being £44 in excess of the previous year's collection.

THE number of cases under treatment during the past year at the Shropshire Eye, Ear, and Throat Hospital, Shrewsbury, was 3,554, of which 589 were in-patients.

INCREASED expenditure, owing to necessary structural improvements and decreased income, have caused a considerable deficit in the funds of the Mildenhall Cottage Hospital.

LORD WANTAGE, V.C., K.C.B., has been elected president of the Royal Berkshire Hospital in place of the late Mr. Richard Beynon, who had occupied that position for 43 years.

AT the St. Paul's Eye and Ear Hospital, Liverpool, the receipts amounted to £1,747, and the expenditure to £1,753. The in-patients numbered 1,171 against 987 in 1896.

THERE were 68 patients treated at the Dartmouth Cottage Hospital in 1897, or 25 more than in the previous year. The expenditure for 1897 amounted to £304, an increase of £61.

DURING 1897, 197 children (as against 182 in 1896) were benefited at the Children's Convalescent Home at Yarmouth. Although 15 more were admitted, the cost of maintenance is £20 less than in 1896.

THE site of the Ruchill Fever Hospital for Glasgow (which was commenced in 1895, and it is expected will be finished in about 12 months) is 32 acres in extent. The hospital will contain over 400 beds.

IT has been proposed by the Bristol County Council to establish a cottage hospital at Avonmouth for the hospital accommodation of workmen injured whilst working at the docks for the Corporation or others.

THE expenditure at the Northallerton Cottage Hospital compares favourably with previous years when the increased number of patients treated is taken into consideration. The expenditure was £617, and the patients numbered 72.

THE patients admitted to the Scarborough Hospital in 1897 show an increase all round over 1896. Thus the in-patients admitted in 1897 were 424, against 390 in 1896; out-patients, 3,132, against 2,872; home patients, 1,340, against 1,312; and casualties, 3,833, against 3,733.

THE average daily cost per in-patient at the Braintree and Bocking Cottage Hospital in 1897 was 3s. 4d., as compared with an average for the previous three years of 3s. 11½d. This makes the cost per patient £4 0s. 8d., to which each patient contributed, on an average, 12s. 10¼d.

THE site for the new branch of the Liverpool Hospital for Consumption at West Kirby having been secured, the plans for the building are in course of preparation. About £50,000 is required for building this branch and rebuilding the hospital at Mount Pleasant, and about £26,000 has so far been subscribed.

THE total outlay on the alterations and improvements at the Stroud General Hospital is expected to reach £1,000. These improvements include a new system of heating by hot water, the introduction of new baths, the repainting of the whole of the hospital used by the patients, the tiling of the lower parts of the walls of the main corridors, improvements in the water supply (hot and cold) and cooking appliances, and alterations to the sanitary arrangements.

SIR JOHN HOLDER has presented to the Birmingham General Hospital a medallion bust of the Queen, in Carrara marble, by Dante Sodini, of Florence. Her Majesty's face is shown in three-quarter view, and she is represented as wearing her widow's cap. The bust will be let into the wall in the out-patient department, between the tablets recording the laying of the foundation-stone of the hospital by the Duke of York and the opening of the building by Princess Christian on behalf of the Queen.

IN order that the drainage and sanitary arrangements of the building should be as good as possible, the governors of the Ashton District Infirmary instructed the North-Eastern Sanitary Association to examine and report upon them. The report of the association was, that whilst the arrangements were as good at the infirmary as they could be expected to be at the time it was built, the present systems and appliances were much more elaborate and effective, and they recommended a completely new system of drainage of all the three buildings—the infirmary, the nurses' home, and the children's hospital. Plans and specifications were prepared by the engineer of the association, and the work is now in hand.

### The Student of Medicine.

### APPOINTMENTS.

Dr. E. B. Allan, appointed Health Officer for Daylesford, Victoria, Australia. W. G. Armstrong, M.B., M.S.Sydney, appointed Medical Officer of Health for the combined Metropolitan Districts, Sydney, New South Wales. K. de Bisley Bell, M.R.C.S., L.R.C.P., appointed House Surgeon to King's College Hospital. H. C. Bennett, M.B.Lond., M.R.C.S., L.R.C.P., appointed Assistant House Surgeon to the Staffordshire General Infirmary, Stafford. P. L. Benson, B.A.R.U.I., M.D., D.P.H.-Camb., appointed Medical Officer of Health to the Bubkingham Rural District Council. J. C. Bowie, M.B., C.M.Aberd., appointed Medical Officer of Health for the Parishes of Sandsting, Aithsting, Walls, and Foula, and Sandness and Papa Stour in the county of Shetland. J. C. Briscoe, M.R.C.S., L.R.C.P., appointed House Physician to King's College Hospital. J. L. Carstairs, M.A., M.B., C.M., appointed Physician for Diseases of Women to the Glasgow Central Dispensary. A. Chenery, M.R.C.S., L.R.C.P., appointed House Surgeon to King's College Hospital. J. Cuthbert, L.R.C.P.Edin., L.R.C.S.I., appointed Health Officer for the Victoria Park District of Sydney, Western Australia. R. Dick, M.B.Sydney, appointed Medical Officer of Health for the combined Hunter River Districts of Newcastle, New South Wales. J. J. FitzGerald, M.D.Brux., L.R.C.P., L.R.C.S.Edin., L.F.P.S.,Glasg., L.A.H.Dub., appointed Fourth Assistant Medical Officer to the Cork District Lunatic Asylum. G. Flux, M.D.Brux., M.R.C.S., L.R.C.P., L.S.A., appointed Assistant Anæsthetist to the Dental Hospital of London. Mr. J. Fuller, appointed Medical Officer of Health to the Long Ashton Rural District Council. T. H. Gardner, M.R.C.S., L.R.C.P. appointed House Physician to King's College Hospital. H. P. Godfrey, F.R.C.S.Eng., M.B.Melb., appointed Health Officer for Bardock, Western Australia. J. W. Hall, L.A.H.Dub., appointed Resident Medical Officer to the Simmer and Jack Hospital, Germistan, South African Republic. F. J. Hasslacher, M.R.C.S., L.R.C.P., appointed House Accoucheur to King's College Hospital. T. A. Hawkeworth, M.R.C.S., L.R.C.P., appointed House Surgeon to King's College Hospital. P. James, F.R.C.S.Eng., appointed a Surgeon to the Wellington Hospital, New Zealand. J. Jeffares, L.R.C.P., M.R.C.S.Edin., L.F.P.S.Glasg., appointed Medical Officer for the Kegworth District of the Shardlow Union. C. T. Lewis, M.R.C.S., L.R.C.P., appointed Assistant House Accoucheur to King's College Hospital. J. H. Marsh, L.R.C.P., L.R.C.S.-Edin., appointed Medical Officer of Health of the Little Hulton Urban District Council. G. H. Parry, M.B., C.M.Edin., appointed Assistant Resident Medical Officer to the Royal National Hospital for Consumption and Diseases of the Chest, Ventnor. A. S. Ransome, M.B., B.C.Camb., D.P.H., appointed Medical Officer of Health to the Southgate Urban District Council. F. R. Sutton, M.B., B.S.Durh., appointed a Resident Surgeon to the Birmingham General Dispensary. F. E. Taylor, M.A., M.B., Ch.B., B.Sc.Vict., M.R.C.S.Eng., L.R.C.P.Lond., appointed House Surgeon to the Hospital for Women and Children, Leeds. R. H. Wilkin, M.R.C.S., L.R.C.P., appointed Medical Officer for the Fifth District of the Thirgoe Union. D. E. Williams, L.R.C.P., L.R.C.S.Irel., appointed Health Officer for Bunbury, Western Australia.

